# Cervical cancer screening service utilization and associated factors among HIV positive women attending adult ART clinic in public health facilities, Hawassa town, Ethiopia: a cross-sectional study

**DOI:** 10.1186/s12913-019-4718-5

**Published:** 2019-11-19

**Authors:** Abiyu Ayalew Assefa, Feleke Hailemichael Astawesegn, Bethlehem Eshetu

**Affiliations:** 1 Department of public health, Hawassa college of health science, P.O.Box: 84, Hawassa, Ethiopia; 2 School of public health, College of medicine and health sciences, Hawassa University, Hawassa, Ethiopia

**Keywords:** Cervical cancer, Cervical cancer screening, Ethiopia, HIV positive women

## Abstract

**Background:**

In Ethiopia, cervical cancer is a public health concern, as it is the second most cause of cancer deaths among reproductive age women and it affects the country’s most vulnerable groups like; rural, poor, and HIV-positive women. Despite the strong evidence that cervical cancer screening results in decreased mortality from this disease, its utilization remains low.

**Methods:**

An institution-based cross-sectional study was conducted from March 2 to April 1/2019 to assess the level and factors affecting utilization of cervical cancer screening among HIV positive women in Hawassa town. Quantitative data collection methods were used. Data were gathered using a structured and pretested questionnaire. Epi-Info version 7 and SPSS version 23 were used for data entry and analysis respectively. Statistically significant association of variables was determined based on Adjusted Odds ratio with its 95% confidence interval and *p*-value of ≤0.05.

**Results:**

Of the 342 women interviewed, 40.1% (95% CI: 35.00, 45.33%) of them were screened. Having a post primary education (AOR = 5.1, 95% CI: 1.8, 14.5), less than 500 cell/mm3 CD4 count (AOR = 2.7, 95% CI: 1.2, 5.9); duration since HIV diagnosis (AOR = 4.2, 95% CI: 2.1, 8.5), partner support (AOR = 4.7, 95% CI: 2.3, 9.4), having knowledge about risk factors (AOR = 2.9 (95% CI: 1.2, 6.9) and having favorable attitude towards cervical cancer and its screening (AOR = 3.7 (95% CI: 1.8, 7.5) were associated with cervical cancer screening utilization.

**Conclusions:**

The study revealed utilization of cervical cancer screening service was low among HIV positive women. Educational status, duration of HIV diagnosis, partner support, knowledge status about risk factor, CD4 count and attitude towards cervical cancer and its screening were associated with cervical cancer screening utilization. Health care workers need to provide intensive counseling services for all ART care attendants to increase utilization.

## Background

Cancer is a disease in which cells in the body grow out of control and when starts at the cervix, it is called cervical cancer(CC) [1]. Seventy percent of all CC cases throughout the world are caused by only two types of human papillomavirus (HPV); HPV-16 and HPV-18 [2]. Many studies revealed that women living with human immunodeficiency virus (HIV) have a higher prevalence of HPV along with infection with multiple high-risk HPV types [3, 4].

According to the World Health Organization (WHO), in areas where HIV is endemic, cervical cancer screening (CCS) results may be positive for precancerous lesions in15–20% of the target population [1]. A comparative study conducted to assess cervical cytology among HIV positive and HIV negative women in a tertiary hospital in north-central Nigeria shows that abnormal Papanicolou Smear (pap smear) results were higher in HIV positive women which is 76 (56.3%) compared with HIV negative women of 17 (12.6%) [5]. Similarly, a study conducted in south Ethiopia, 22% of women infected with HIV were positive for precancerous lesions [6]. Global cancer statistics indicate that CC ranks fourth for both incidence (6.6%) and mortality (3.5%) among females in 2018 [7]. About 85% of new cases and 87% of all deaths of CC occur in the less developed regions [8].

In Ethiopia, CC is a public health concern, as it is the second most cause of cancer deaths among women aged 15 to 44 years next to breast cancer [9]. As a major public health burden, it affects the country’s most vulnerable groups like; rural, poor, and HIV-positive women [3]. According to international agency for research on cancer information center on HPV and Cancer of Ethiopia, current estimates indicate that every year 7095 women are diagnosed with CC and 4732 die from the disease [9]. In Ethiopia, CC is the most common (31.8%) diagnosed cancer among all cancer cases and having increasing fashion [10]. Due to the fact that there is lack of information about CC and prevention services, majority of CC (over 80%) in Sub-Saharan Africa are detected in late stage which is associated with low survival rates after surgery or radiotherapy [11].

Ethiopia, being a developing country, has adopted cheaper but effective techniques for screening of CC called Visual Inspection with Acetate (VIA). Pathfinder International Ethiopia has implemented single visit approach of VIA screening and cryotherapy of precancerous lesions for HIV positive women under the project name of “Addis Tesfa “CC prevention project from October/2009 – September/2014 [4]. Well organized programs to detect and treat precancerous abnormalities at the early stages of cancer prevent up to 80% of CC in developed countries [12]. However, in low- and middle-income countries approximately 5% of eligible women undergo cytology-based screening in a 5 year period which is an obstacle to prevent the occurrence of CC [13].

Federal ministry of health targeted to achieve at least 80 % coverage of the appropriate target populations with screening and treatment for pre-invasive cervical-cancer cases by 2020 [14]. However, a Community-based Cross-sectional survey of nine regions and two city administrations (Addis Ababa and Dire Dawa) of Ethiopia shows extremely low rate of cervical screening (2.9%) [15] and only 10% of patients have come to the oncology center in early stage I and II [16]. Despite having started cervical cancer screening (CCS) service the evidence of utilization among HIV positive women is not known in the study area. Therefore, this study aimed to assess the utilization of CCS and associated factors among HIV positive women in public health facilities, Hawassa town.

## Methods

### Study design and setting

An institution-based cross-sectional study was conducted from March 2 to April 1/ 2019 in Hawassa town. Hawassa town is located 275 Km to the south of Addis Ababa (the capital city of Ethiopia) on the shoreline of Lake Hawassa. Only, three public health facilities (Adare general hospital, millennium health center, and hawassa referral hospital) provide both CCS and antiretroviral therapy (ART) services in the town.

### Source population

All HIV positive women attending adult ART Clinics at Public health facilities with CCS service in Hawassa town.

### Study population

The study populations were selected HIV positive women attending adult ART clinic in public health facilities with CCS service during the study period.

### Sample size determination

The sample size was calculated using two population proportions by taking more frequently observed associated factors for the utilization of CCS, like age, positive perception and diagnosed for HIV [17, 18]. Epi info Stat Calc functions were used to compute the sample size; accordingly, it became 271, 257 and 350, respectively. Finally, the larger sample size (350) was selected.

### Sampling procedure

From all public health facilities, all facilities that provide both CCS and ART service (Hawassa University Specialized Comprehensive Hospital, Millenium Health Center and Adare General Hospital) were included. The number of study participants to be included in each facility was determined in proportion with the total number of women who came to the ART services, using estimated patient flow of 6 months before the data collection. Using a systematic random sampling technique, every second woman on the list of their order of arrival for follow up care was selected and formed the participants of the study.

### Data collection

A structured interviewer-administered questionnaire was used to collect relevant information from each study respondent. The questionnaire was prepared by reviewing different related literature [17–20] with modification in line with the objectives of this particular study and was prepared in English and then translated to Amharic and local language (Sidaamu Afoo).

The questionnaire was pre-tested on 5% of the total sample size in nearby hospital (Yirgalem hospital). Data collectors and principal investigators were involved during the pretest. Based on the pretest, appropriate modifications were made before the actual data collection.

The final questionnaire had six parts: social demographic and economic factors, Knowledge (risk factors, symptoms, preventive method, ways of screening methods of CC and importance of CCS), medical and reproductive health characteristics, attitude towards CC and screening, screening practice questions and health service-related questions. Five data collectors and one BSc nurse from Hawassa comprehensive specialized hospital supervised the data collection process. Data were collected using face to face exit interviews during government working hours at selected health facilities.

### Variable definitions

#### Utilization of CCS

HIV positive women who were screened for premalignant cervical lesions at least once within 5 years of data collection period [13].

#### Knowledge

knowledge about risk of CC was considered good if a respondent mentioned at least 3 from listed 6 known risk factors, otherwise indicated as having poor knowledge about the risk of CC. Knowledge about symptoms of CC was considered good if a respondent mentioned at least 3 from listed 6 known symptoms, otherwise indicated as having poor knowledge about symptoms of CC. Knowledge about ways of screening of CC was considered good if a respondent mentioned at least 1 of the known technique otherwise indicated as having poor knowledge about ways of screening of CC. Knowledge about prevention methods of CC was considered good if a respondent mentioned at least 3 from listed 6 known prevention methods, otherwise indicated as having poor knowledge about prevention methods of CC.

Knowledge about the benefit of CCS was considered good if a respondent mentioned at least 2 from listed 4 known benefits, otherwise indicated as having poor knowledge about the benefit of CCS [19].

Attitude: Attitude towards CC and its screening was measured using different attitude questions and woman answered equal to and above the mean value was considered having favorable attitude and woman answered below the mean value was considered having unfavorable attitude [21].

### Data management and data analysis

Data were entered into Epi-info version 7 and imported to Statistical Packages for Social Sciences (SPSS) version 23 for analysis. The dependent variable was the utilization of screening for CC and assigned 1 when a respondent reported to have ever been screened and 0 when otherwise. The data analysis ranged from the basic description to the identification of factors that are associated with CCS utilization. Both bivariable and multivariable logistic regression models were fitted to identify factors associated with CCS service utilization. Crude and Adjusted Odds ratio with 95% confidence interval were computed to determine the level of significance. In the bivariate analysis, variables that had a significant association with the outcome variable at less than 0.2 *p*-values were considered for multivariable analysis [18]. Finally, a statistically significant association of variables was determined based on the Adjusted Odds ratio with its 95% confidence interval and a *p*-value of < 0.05 [17, 19]. The Multivariable model was tested for goodness of fit with the Hosmer Lemeshow test and it is non-significant. The results were presented using tables, graphs, and charts.

## Results

### Socio-demographic characteristics of respondents

A total of 342 (97.7%) mothers were included in this study. The mean age (+/−standard deviation) of the respondents was 33.4 years (+/− 8.7 years). One hundred seventy-four (50.9%) of the respondents had two and above children. Most of the respondents149 (43.6%) had acquired post-primary level education. Majority of the respondents (88%) were urban dwellers and 119 (34.8%) of respondents were self-employed. Most of the respondents 185 (54.1%) reported having less than 1000 Ethiopian birr of average monthly income. Regarding marital status; more than half 242 (70.8%) of respondents were ever married **(**Table 1**).**

**Table 1:** Socio-demographic characteristics of HIV positive women attending adult ART clinic in Public Health Facilities, Hawassa town, Ethiopia, 2019

Variable	Screened for cervical cancer		Total
	Yes	No	
Age			
< = 24	21	36	57
25–29	24	72	96
30–34	14	20	34
35–39	39	36	75
> = 40	39	41	80
Residence of respondents			
Urban	124	177	301
Rural	13	28	41
Parity			
< 2	55	113	168
≥ 2	82	92	174
Educational level			
No formal education	10	51	61
Primary	28	104	132
Post primary	99	50	149
Marital Status			
Never Married	37	63	100
Ever married	100	142	242
Occupational Status			
Unemployed	4	15	19
Civil servant	40	21	61
Private employ	18	39	57
Self employed	47	65	112
House wife	17	38	55
Daily labor	9	22	31
Others^@^	2	5	7
Average monthly income (Ethiopian birr)			
≤ 1000	53	132	185
1001–2000	39	37	76
≥ 2001	45	36	81

@ = Commercial sex worker [3], Student [3] and Farmer [1]

### Knowledge about cervical cancer and its screening among respondents

**Fig. 1:**
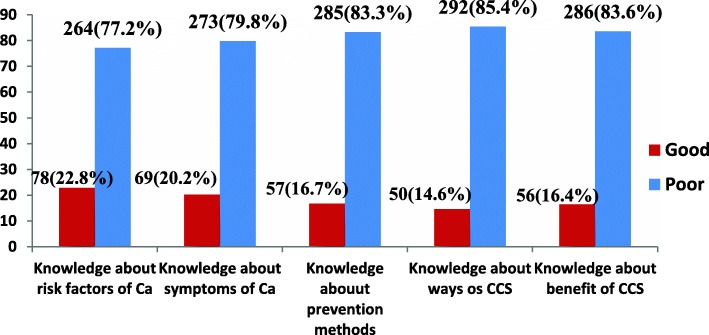
Specific knowledge status of HIV positive woman attending adult ART clinic in Public Health Facilities of Hawassa town, Ethiopia, 2019. Ca: Cervical cancer; CCS: Cervical cancer screening

One hundred twenty-nine (37.7%) respondents stated having sexually transmitted infections increases the risk of one developing CC. But some of the respondents 87(25.4%) didn’t know the risk factors of CC. Our finding also revealed that 140 (40.6%) respondents mentioned that pelvic pain is signs and symptoms of CC whilst almost one-third of respondents 130 (38.0%) didn’t know the signs and symptoms of CC. Nearly half of respondents 161(47.1%) mentioned CCS followed by respondents who cited consistent condom use 114 (33.3%) as prevention methods of CC and a considerable number of respondents 88(25.7%) didn’t know any prevention methods of CC. The majority of respondents, 292(85.4%) didn’t know CCS technique. Two hundred seventeen (63.5%) respondents mentioned early detection as the benefit of CCS while very few numbers of respondents 4(1.2%) cited decreasing chance of abortion **(**Table 2**).** When we see the overall knowledge status of respondents, seventy-eight 78 (22.8%) women mentioned three or more risk factors of CC correctly whereas only 57 (16.7%) knew three or more prevention methods of CC **(**Fig. 1**).**

**Table 2:** Knowledge items responses of HIV positive women attending adult ART clinic in Public Health Facilities, Hawassa town, Ethiopia, 2019

Knowledge items		Frequency	Percentage
Risk factors	Unsafe sexual practice	132	38.6
	Sexually transmitted infections	129	37.7
	Having multiple sexual partner	97	28.4
	Smoking	70	20.5
	Early sexual activity	49	14.3
	Prolonged use of oral contraceptive	11	3.2
	Do not know	87	25.4
Sign and symptoms of cervical cancer	Pelvic pain	140	40.9
	foul smelling vaginal discharges	136	39.8
	post coital bleeding	74	21.6
	Lengthy menstruation	46	13.5
	pain during sex	24	7.0
	Inter menstrual bleeding	11	3.2
	Do not know	130	38.0
Prevention methods	Cervical cancer screening	161	47.1
	Consistent condom use	114	33.3
	Treatment of STIs	62	18.1
	Reduce sexual partner	59	17.3
	Late marriage	31	9.1
	Vaccination	12	3.5
	Do not know	88	25.7
Benefit of screening	Early detection	217	63.5
	Early treatment	89	26.0
	Early diagnosis	62	18.1
	Decreasing chances of an abortion	4	1.2
	Do not know	25	13.2

### Attitude of respondents towards cervical cancer and its screening

Above half of respondents, 182(53.2%) strongly disagree with the chance of getting the disease. The respondents were asked whether having CCS results in one being infertile or not. Forty-seven (13.7%) of the respondents strongly agreed for the positive effect of CCS for infertility; 72(21.1%) were not sure whether CCS could result in infertility, but 194(56.7%) of the respondents disagreed strongly to this question. Some of the respondents, 110(32.2%) disagreed strongly with the notion that it was important for women to have CCS even if they do not make sexual act while 101(29.5%) agreed strongly **(**Table 3**).** From a total of 342 respondents, 161 (47.1%) have a favorable attitude towards CCS.

**Table 3:** Responses of attitude questions towards cervical cancer and its screening among HIV positive women attending adult ART clinic in Public Health Facilities, Hawassa town, Ethiopia, 2019

Items	Strongly disagree (%)	Disagree (%)	Not sure (%)	Agree (%)	Strongly agree (%)
Do you believe chance of getting the disease?	182(53.2)	37(10.8)	40(11.7)	38(11.1)	45(13.2)
Is CCS undertaken only when there is symptom?	206(60.2)	13(3.8)	30(8.8)	22(6.4)	71(20.8)
Does it important undertaking CCS even if you does not make sexual act?	110(32.2)	21(6.1)	89(26)	21(6.1)	101(29.5)
Is cervical cancer is more serious than other disease?	47(13.7)	10(2.9)	30(8.8)	20(5.8)	235(68.7)
Do you believe cervical cancer screening is painful?	139(40.6)	24(7)	74(21.6)	34(9.9)	71(20.8)
Do you believe cervical cancer screening may cause infertility?	194(56.7)	10(2.9)	72(21.1)	19(5.6)	47(13.7)

### Medical and reproductive characteristics of respondents

According to medical records, documentation mean baseline Cluster of Differentiation 4 (CD4 count) of the respondents was 422 cells/mm3 (SD = 281.3). Just 135(39.5%) of women were in WHO clinical stage I. The majority of women, 261(76.3%) claimed that they had no history of multiple sexual partners and twenty-two (6.4%) of them had family history of CC. Our study demonstrated that a majority of respondents 207 (60.5%) had got recommendation for CCS by health professional. Most women 211(61.7%) had not gain partner or husband support to check their gynecological health **(**Table 4**).**

**Table 4:** Medical and reproductive characteristics of HIV positive woman attending adult ART clinic in Public Health Facilities, Hawassa town, Ethiopia, 2019

Variables	Frequency	Screened for cervical cancer	
		Yes	No
Diagnosed for HIV (year)			
< 5	164	40	124
≥ 5	178	97	81
WHO clinical stage			
One	135	32	103
Two	83	34	49
Three	68	42	26
Four	56	29	27
CD4 count (cell/mm^3^)			
< 500	227	116	111
≥ 500	115	21	94
Duration of enrollment			
< = 4	147	36	116
5–9	109	56	51
> 10	86	45	38
Multiple sexual partner			
No	261	99	162
Yes	81	38	43
Family history of cervical cancer			
No	320	120	200
Yes	22	17	5
Age at first sexual act			
≤ 20	134	49	85
> 20	208	88	120
Partner support			
Yes	131	81	50
No	211	56	155
Recommendation by provider for cervical cancer screening			
Yes	207	87	120
No	135	50	85

### Utilization of cervical cancer screening among HIV positive women

**Fig. 2:**
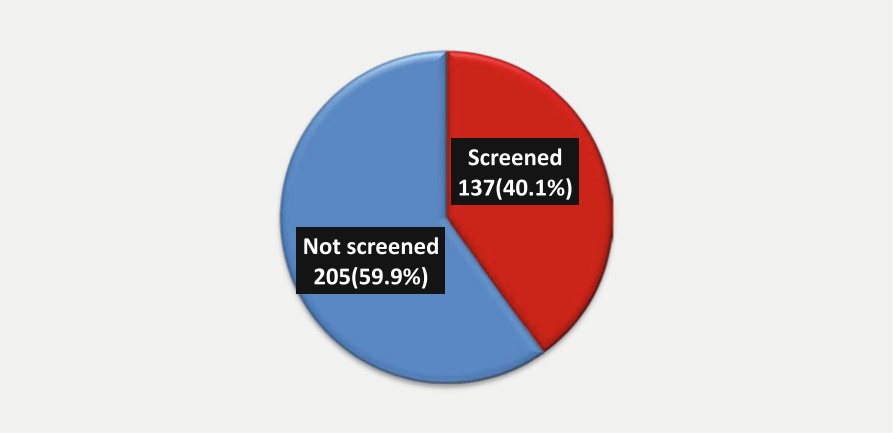
Cervical cancer screening utilization among HIV positive women attending adult ART clinic in Public Health Facilities of Hawassa town, Ethiopia, 2019

**Fig. 3:**
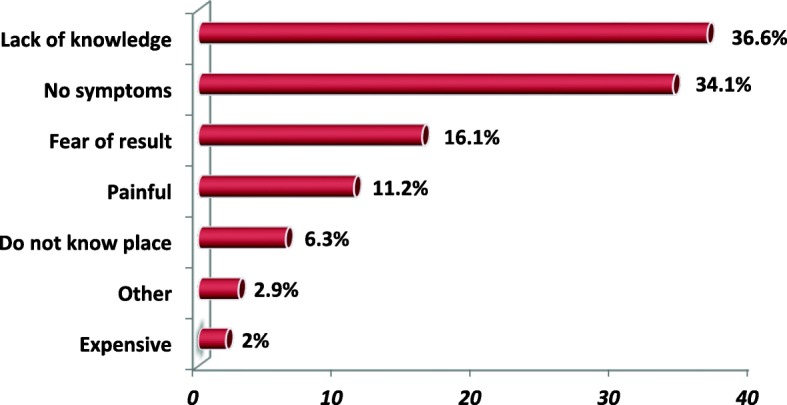
Reason for not-screened among HIV positive women attending adult ART clinic in Public Health Facilities of Hawassa town, Ethiopia, 2019. Other: culture, lack of time [2], feeling not susceptible, no reason [2]

In this study, 137 (40.1%) [95% CI: 35.00, 45.33%] of the respondents were found screened within the past 5 years whilst, 205 (59.94%) [95% CI: 54.67, 65.00%] of them did not **(**Fig. 2**).** Out of 205 who did not screen, seventy-five (36.6%) reported that lack of knowledge about CC and its screening is the major reason for not undertaking CCS. Other reasons were lack of symptoms, fear of test result, having thought of CCS is painful, and not knowing the place where CCS is performed, etc. **(**Fig. 3**).**

### Factors associated with CCS utilization among adult HIV positive women attending ART clinic

During bivariable Logistic regression from 18 variables, 13 of them met the criterion to be included into multivariable logistic regression by yielding a *p*-value of < 0.2. These are partner support, WHO clinical stage, attitude towards CC and its screening, CD4 count, average monthly income, duration since HIV diagnosis, educational status, knowledge about risk factors, knowledge about prevention of CC, knowledge about benefit of screening, parity, having multiple sexual partner and age of respondent. After multivariable analysis was conducted, six variables (partner support, attitude towards CC and its screening, CD4 count, duration since HIV diagnosis, educational status, and knowledge about risk factors) remained significant with a *p*-value of less than 0.05.

Educational status has a significant association with CCS utilization. The odd of being screened among respondents having post-primary education were 5.1 times (AOR = 5.1, 95% CI: 1.8, 14.5) higher as compared to respondents who have no formal education.

The odds of CC screening service utilization among respondents whose HIV diagnosis was made 5 years or more (≥ 5 years) back were four times (AOR = 4.2, 95% CI: 2.1, 8.5) compared to whose HIV diagnosis was made less than 5 years (< 5 years). Women who had less than 500 cell/mm3 CD4 count were three times (AOR = 2.7, 95% CI: 1.2, 5.9) more likely to be screened compared to women who had greater than or equal to 500 cells/mm3 CD4 count.

The odds of getting screened for CC among HIV positive women having partner support were 4.7 times (AOR = 4.7, 95% CI: 2.3, 9.4) higher than those who had no partner support. Women who have favorable attitudes toward CC and its screening were 3.7 times more likely to utilize CCS service (AOR = 3.7 (95% CI: 1.8, 7.5). Keeping all other factors constant, the chance of CCS were three times higher for those respondents having good knowledge about risk factors of CC (AOR = 2.9 (95% CI: 1.2, 6.9) than their counterparts **(**Table 5**).**

**Table 5:** Multi-variable analysis of selected variables with utilization of cervical cancer screening among HIV positive women attending adult ART clinic in Public Health Facilities, Hawassa town, Ethiopia, 2019

Variables	Screened for cervical cancer		COR (95% CI)	AOR(95% CI)
	Yes	No		
Age in years				
< = 24	21	36	1	1
25–29	24	72	0.6 (0.3, 1.2)	0.6 (0.2, 1.7)
30–34	14	20	1.2 (0.5, 2.9)	1.6 (0.4, 6.2)
35–39	39	36	1.9 (0.9, 3.8)	1.7 (0.5, 5.6)
> = 40	39	41	1.6 (0.8, 3.3)	0.9 (0.3, 2.9)
Educational status				
No formal education	10	51	1	1
Primary	28	104	1.4 (0.6,3.0)	1.3 (0.46, 3.7)
Post primary	99	50	10.1 (4.7, 21.6)	5.1 (1.8, 14.5)*
Parity				
< 2	55	113	1	1
≥ 2	82	92	1.8 (1.2, 2.8)	1.5 (0.7, 3.3)
Average monthly income (ETB)				
≤ 1000	53	132	1	1
1001–2000	39	37	2.7 (1.5, 4.6)	1.5 (0.7, 3.4)
≥ 2001	45	36	3.1 (1.8, 5.4)	1.6 (0.7, 3.9)
WHO clinical stage				
One	32	103	1	1
Two	34	49	2.2 (1.2, 4.0)	1.3 (0.6, 3.1)
Three	42	26	5.2 (2.8, 9.8)	1.5 (0.6, 4.1)
Four	29	27	3.5 (1.8, 6.7)	2.1 (0.8, 5.9)
CD4 count (cell/mm^3^)				
< 500	116	111	4.7 (2.7, 8.0)	2.7 (1.2, 5.9)*
≥ 500	21	94	1	1
Time since diagnosis of HIV (yrs.)				
< 5	40	124	1	1
≥ 5	97	81	3.7 (2.3, 5.9)	4.2 (2.1, 8.5)*
Multiple sexual partner				
Yes	38	43	1.5 (0.9, 2.4)	1.6 (0.7, 3.5)
No	99	162	1	1
Partner support				
Yes	81	50	4.5 (2.8, 7.2)	4.7 (2.3, 9.4)*
No	56	155	1	1
Knowledge about risk factors of Ca				
Good	51	18	6.8 (3.8, 12.0)	2.9 (1.2, 6.9)*
Poor	86	187	1	1
Knowledge about prevention method				
Good	42	15	5.6 (3.0, 10.6)	2.6 (0.9, 7.4)
Poor	95	190	1	1
Knowledge about benefit of screening				
Good	34	22	2.7 (1.5, 4.9)	1.7 (0.7, 4.2)
Poor	103	183	1	1
Attitude toward Ca and its screening				
Favorable	101	60	6.8 (4.2, 11.0)	3.7 (1.8, 7.5)*
Unfavorable	36	145	1	1

*P-value: < 0.05, *ETB* Ethiopian birr, *COR* Crude odd ratio, *AOR* Adjusted odd ratio, *CI* Confidence Interval, 1: Reference category

## Discussion

In our study, we have found that 40.1% (95% CI: 35.00, 45.33%) of HIV positive women utilize CCS, which is significantly higher compared with the study done in Morocco (9%) [22], Gondar (10%) [18] and Addis Ababa (11.5%) [19]. It is also higher compared with studies conducted in Uganda [23] and Gondar [17] having screening prevalence of 30.3 and 23.5%, respectively. This might be due to the improved expansion and access of screening centers especially after the start of VIA in many health facilities, the enhanced nation-wide advocacy, media concern, community sensitization and awareness creation through expansion of urban health extension program about the CCS that has been put into effect in recent years (time difference).

However, this finding is lower than the study findings in Canada [24] 58%, England [25] 85.7%, Catalonia [26] 50.6%, and Kenya [27] 46%. The possible reason for this variation could be due to differences in socio-demographic and economic status of the study respondents as well as the countries’ promotional policy variations. Another reason for decreased screening utilization may be due to uneven distribution of screening services centers. For example; there is universal access to health care in Canada, including the availability of primary care and specialist physicians, which differs from other health care models [24]. Similarly, Kenya has a more robust CCS program; as a result, there is increased awareness about CC and its screening [28].

According to the reports of our study, the main reason cited for not undergoing CCS was lack of knowledge followed by the absence of symptoms. A similar reason was also reported from the study done in India [29] and Gondar [17] which reported a lack of knowledge and no symptoms were among reasons for refusing CCS respectively. Furthermore, absence of symptoms was cited by woman for not undergoing CCS service utilization in a study conducted in Morocco [22].

The findings of our study suggest that educational level has a positive effect on the utilization of CCS service. That is, women who had post-primary level of education are more likely to use CCS services than those with no formal education levels. The same finding was observed in studies done in, India [29], Nigeria [30], Ghana [31], Gondar [18] and Addis Ababa [19], in which level of education can predict CCS. Similarly, a study conducted in Italy [32] reported lack of Pap-smear in the last year was significantly associated with lower educational level. This is not surprising as we expect those women who are educated to have an understanding of the cause, risk factors, prevention mechanism and treatment of the disease and as such can demand screening services. Also, better-educated women have a higher efficiency in the production of health and education as well as impart self-efficacy, confidence, motivation and social inclusion, in search for health interventions. Additionally, education is also believed to facilitate the assimilation of health education given to women in health institutions on common acute and chronic illnesses.

We found that the chance of CCS was about nearly five times higher for those respondents who have partner support compared to respondents who have no partner support. Previous studies done in England [25], Tanzania [33] and Kenya [34], also have linked partner support with increased CCS service utilization. This could be explained by the fact that the active involvement of male partners makes them more aware of the significance of maternal health care services and support their partners. However, this finding contradicts the findings of a study conducted in India which revealed that their husband did not allow them to utilize CCS service [35]. This might be due to the difference in the study site. For example, 54.8% of respondents were from rural setting in the study done in India but 88% respondents of this study were from urban setting which indirectly indicating the awareness level of their partner might be a contributing factor.

Again our study demonstrated that women’s having CD4 count less than 500 cell/mm3 were 2.7 times more likely to be screened for CC than those who had more than or equal to 500 cell/mm3. This finding is in agreement with the study done in Gondar [18] that CD4 count of </=200 cell/mm3 was significantly associated with cervical screening. The possible explanation could be women with lower CD4 count might have decreased immunity which intern leads to the development of opportunistic infections which increases the development of signs & symptoms of disease and visiting of health facility. This frequent health facility attendance can be viewed as an opportunity to provide health education and screening service for CC. Evidence showed that immune-suppression and low CD4 counts caused by HIV infection predisposes women living with HIV infection at an increased risk for CC and the development of squamous intraepithelial lesions [36, 37]. Additionally, different scholars have shown that the health-seeking behavior of individuals is better during symptomatic illness than if there is no symptom [38]. But a study in Northern Italy [32] is not similar to our study; it shows HIV positive women with CD4 count of < 200 cell/mm3 were more likely to have no history of Pap-smear in the year before the questionnaire. This can be due to access to screening at an early stage of illness without delay unlike developing countries like Ethiopia, many investigations will be offered for patients when they are seriously ill.

From this study, it was found that the length of time since HIV diagnosis is one of the significant predictors of CCS service utilization. The odds of CC screening service utilization among respondents whose HIV diagnosis was made five or more years back were four times higher than those whose HIV diagnosis was made before 5 years (< 5 years). Comparable findings also reported from a study done in Italy [32], Kenya [28] and Gondar [18]. This might be related to the fact that the higher the number of years’ of HIV diagnosis, the higher the probability for them to get interacted with health care providers who are the main source of information about CCS for this study respondents, and the greater exposure to cancer related information, which might in turn resulted in increased use of screening services for CC.

This study also revealed that knowledge about risk factor is significantly associated with CCS service utilization. i.e. Those with good knowledge about risk factors of CC are above three times more likely to be screened than those with poor knowledge about risk factors of CC. This finding is similar to studies done in England [25], Japan [39] and China [40]. This might be because of HIV positive women having good knowledge about the risk factors of CC might have good health-seeking behavior.

Finally, our study found that having had a favorable attitude towards CC and its screening had been associated with an increased use of screening services. This finding is shared with a previous study conducted in Nigeria [41] which demonstrated that respondents who had negative attitude had 63% lesser odds of being screened compared to those who had positive attitudes towards screening. Similarly, other previous studies conducted in Ethiopia, like in Mekelle [42], Gondar [17], and Finote Selam [21] also support our finding by reporting positive association of having a positive attitude with utilization of CCS. The reason might be having a favorable attitude is mostly followed by having an understanding of the cancer of cervix and engagement in cervical screening as well. Additionally, this was consistent with the hypothesis of the Health Belief Model stated that perceived severity and threat of CC, perceived benefit, perceived self-efficacy and net benefit about the preventive action of CC and its screening necessitate people to engage in preventive actions like CCS service utilization [43]. The possible limitation of this study could be the difficulty of interpreting the findings from a cause and effect relationship as the study is cross-sectional. Few screening status were assessed based on self-report. As a result, the level is likely to be overestimated due to social desirability bias.

## Conclusion and recommendation

Utilization of CCS among HIV positive women in the study area was 40.1%. Even if it remained lagging from recommended coverage of the target group by the national guideline of Ethiopia it is promising when compared to previous studies conducted in Ethiopia. Partner support, attitude towards CC and its screening, time since HIV diagnosed, Educational level, CD4 count and Knowledge about risk factors of CC were significantly associated with utilization of the service among HIV positive women. To mitigate those problems, there should be consistent monitoring on the detection of CC and prevention activities, conducting reproductive health education to modify women’s attitude toward CCS for HIV positive women in every clinical contact. Health care providers should promote partner involvement in maternal health care service including CCS.

## Data Availability

Data is not available for online access, however, readers who wish to gain access to the data can write to the corresponding author Abiyu Ayalew at abiyman143@gmail.com.

## Abbreviations

AOR: Adjusted Odd Ratio
ART: Antiretroviral Therapy
CC: Cervical cancer
CCS service: Cervical Cancer Screening service
CCS: Cervical Cancer Screening
CD4: Cluster of Differentiation 4
CI: Confidence Interval
HIV: Human Immunodeficiency Virus
HPV: Human Papilloma Virus
PAP smear: Papanicolou Smear
VIA: Visual Inspection using Acetic acid
WHO: World Health Organization.
